# Acid Neutralizing Ability and Shear Bond Strength Using Orthodontic Adhesives Containing Three Different Types of Bioactive Glass

**DOI:** 10.3390/ma9030125

**Published:** 2016-02-24

**Authors:** Song-Yi Yang, Seong-Hwan Kim, Se-Young Choi, Kwang-Mahn Kim

**Affiliations:** 1 Department and Research Institute of Dental Biomaterials and Bioengineering, Yonsei University College of Dentistry, Seoul 03722, Korea; syyang88@yuhs.ac; 2 BK21 PLUS Project, Yonsei University College of Dentistry, Seoul 03722, Korea; 3 Department of Materials Science and Engineering, Yonsei University, Seoul 03722, Korea; shkim710@yonsei.ac.kr (S.-H.K.); sychoi@yonsei.ac.kr (S.-Y.C.)

**Keywords:** 45S5 bioactive glass, 45S5F bioactive glass, S53P4 bioactive glass, acid neutralizing ability, shear bond strength

## Abstract

The objective of the study was to compare the acid neutralizing ability and shear bond strength (SBS) of three different types of orthodontic adhesives containing bioactive glasses (BAGs). 45S5, 45S5F and S53P4 BAGs were prepared using the melting technique and ground to fine particles. Orthodontic adhesives containing three types of BAGs were prepared as follows: 52.5% 45S5 BAG + 17.5% glass (45S5_A); 61.25% 45S5 BAG + 8.75% glass (45S5_B); 52.5% 45S5F BAG + 17.5% glass (45S5F_A); 61.25% 45S5F BAG + 8.75% glass (45S5F_B); 52.5% S53P4 BAG + 17.5% glass (S53P4_A); 61.25% S53P4 BAG + 8.75% glass (S53P4_B); and 70.0% glass (BAG_0). To evaluate the acid neutralizing properties, specimens were immersed in lactic acid solution, and pH changes were measured. SBS was measured with a universal testing machine. For all of the BAG-containing adhesives, the one with 61.25% of BAG showed a significantly greater increase of pH than the one with 52.5% of BAG (*p* < 0.05). Groups with 61.25% of BAG showed lower SBS than samples with 52.5% of BAG. 45S5F_A showed no significant difference of SBS compared to BAG_0 (*p* > 0.05). The adhesive containing 61.25% of 45S5F BAG exhibited clinically acceptable SBS and acid neutralizing properties. Therefore, this composition is a suitable candidate to prevent white spot lesions during orthodontic treatment.

## 1. Introduction

Demineralization of enamel around orthodontic brackets, commonly known as white spot lesions (WSLs), often may result in a complications during orthodontic treatment. The demineralized enamel lesions are not only unaesthetic and unhealthy, but also potentially irreversible [1]. Major reasons for the occurrence of these lesions are due to inadequate patient skills and self-care, which are related to the difficulty of cleaning the causative bacterial during tooth brushing, especially in the case of young patients, such as pre-adolescents [2,3].

Orthodontic materials, such as brackets, arch wires, ligature ties and elastomeric rings, lead to rapid accumulation of dental plaque and the accompanying elevated levels of cariogenic bacteria during orthodontic treatment with fixed appliances [4,5]. Cariogenic bacteria surrounding orthodontic appliances produce metabolized acids from fermentable carbohydrate and lead to the reduction of the local plaque pH, which consequently causes the release of calcium and phosphate ions from the enamel apatite [6]. In addition, a recent study reported that the microleakage between the adhesive-enamel interfaces around the bracket base contributes to the invasion of cariogenic bacteria and oral fluids, which may initiate WSLs under the bracket surface [7]. The results of these studies showed that orthodontic adhesive materials are one of the potential risk factors for tooth demineralization during orthodontic treatment.

Bioactive glass (BAG) is reported to be able to stimulate bone regeneration more than other bioactive ceramics. Because of these properties, BAG has been used to repair bone defects in the jaw and has been used as implant materials in orthopedics during the past few decades [8]. Furthermore, it possesses a remineralization effect on the demineralized tooth structure by releasing calcium and phosphate ions, as well as antibacterial and acid neutralizing properties through the increase in pH by the release of alkali ions during dissolution of BAG in an aqueous environment [9,10,11]. In this regard, in dentistry, BAGs have been studied for the augmentation of alveolar ridges, the treatment of periodontal pocket, a filler component of dental materials, such as restorative materials, cements, pit and fissure sealants, and also for the treatment of hypersensitive teeth [12,13,14,15].

In addition to the above, some authors have recently reported that pit and fissure sealants containing 45S5 BAG filler can cause acid neutralization in a cariogenic environment [14]. However, despite the advantages of BAG in terms of the acid neutralizing effect and potential for application during orthodontic treatment to prevent WSLs, there has been a lack of studies investigating such applications. Previous investigations that have introduced the BAG-containing orthodontic adhesive resins showed significantly higher calcium and phosphate ion release, buffering capacity and prevention of WSLs adjacent to brackets, but were limited by considering just one type of BAG or such application [16,17].

However, there have been many variations on the composition of bioactive glass. 45S5 BAG was widely known for its antimicrobial effect, as well as apatite formation in simulated body fluid [18]. Among these, 45S5F was well known for its antimicrobial effect, apatite formation and fluoride releasing, the ability of which is currently required in dentistry [19]. Moreover, S53P4 was also known for the antimicrobial effect and mineralization of dentin [20]. These three types of BAGs are approved by the FDA for clinical applications and have been widely used as bone graft materials [21]. Thus, in the present study, to the best of our knowledge, our study is the first to attempt to find the most efficient orthodontic adhesive containing BAG to prevent the WSLs using three selected BAGs: 45S5, 45S5F and S53P4.

Therefore, the aim of this study was to evaluate the possibility of utilizing the novel materials: three different types of orthodontic adhesives containing BAGs (45S5 BAG, 45S5F BAG and S53P4 BAG) in terms of the acid neutralizing property and shear bond strength with enamel. Commercially available orthodontic adhesive was used as a reference for each of the evaluations. The null hypothesis of this study is that there will be no significant differences in the acid neutralizing property and shear bond strength among the three different types of BAGs.

## 2. Results

### 2.1. Powder Characterization of Three Types of BAG

**Figure 1:**
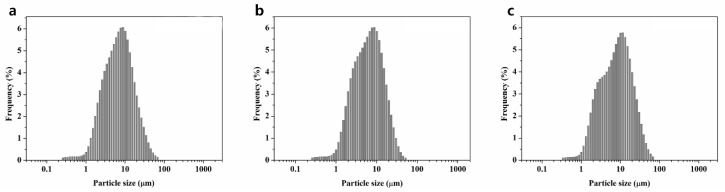
Particle size distributions for three different types of bioactive glass (BAG): (**a**) 45S5 BAG particles; (**b**) 45S5F BAG particles; and (**c**) S53P4 BAG particles.

**Figure 2:**
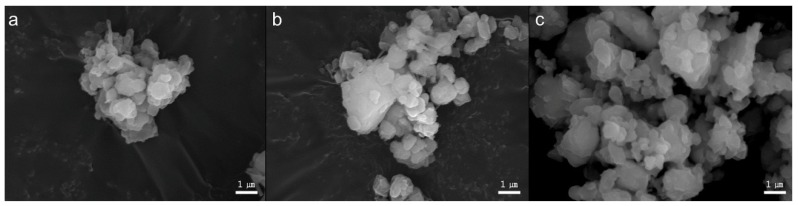
The morphology for three different types of BAG: (**a**) 45S5 BAG particles; (**b**) 45S5F BAG particles; and (**c**) S53P4 BAG particles.

**Figure 3:**
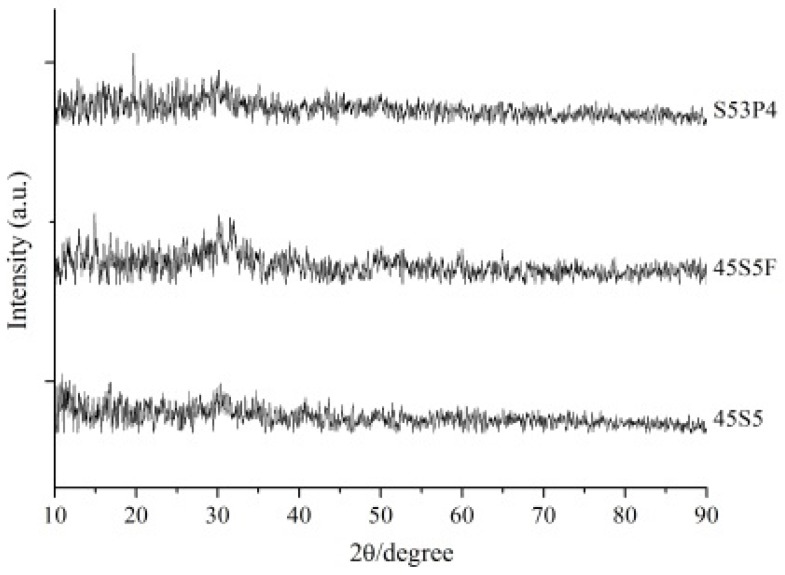
The XRD patterns for three different types of BAG.

Histograms of three different types of BAGs’ particle distribution are shown in Figure 1. The 45S5 BAG particle size ranged from 0.3 to 67.5 µm, with a median value of 6.5 µm. The 45S5F BAG particle size ranged from 0.3 to 51.5 µm, with a median value of 5.9 µm. Finally, the S53P4 BAG particle size ranged from 0.3 to 67.5 µm, with a median value of 7.5 µm. All BAG particles’ morphology was irregular aggregate shapes (Figure 2). Furthermore, all glasses were in an amorphous state, as identified by XRD examination (Figure 3).

### 2.2. Acid Neutralizing Ability

**Figure 4:**
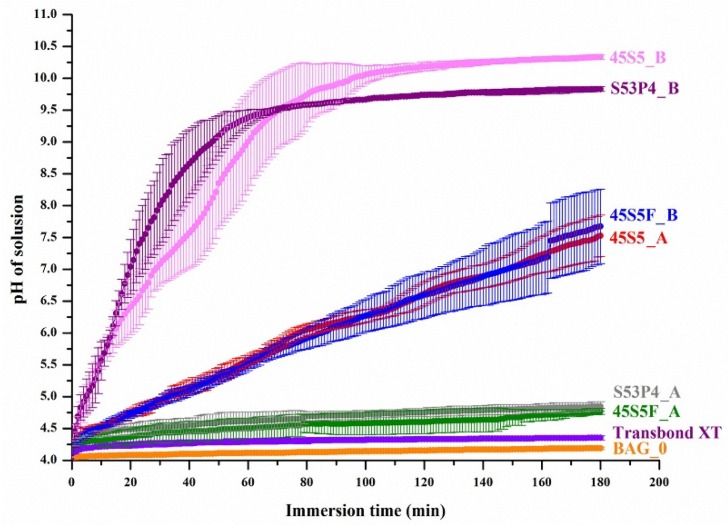
Time-dependent changes of the pH in acid solution.

Figure 4 shows the pH changes of the lactic acid solution in which the specimens were immersed (mean ± SD, *n* = 5). The pH curves of the lactic acid solution for BAG_0 and Transbond XT, which had no BAG, were similar to each other, as both showed a slight increase in pH for the initial 3 min and showed a final pH value below 4.25 after 180 min. In contrast, the BAG-containing experimental groups showed a pH rise during immersion in the lactic acid solution. Each BAG group that contained from 52.50% to 61.25% of BAG exhibited increasing final pH values as more of the BAG was added regardless of the BAG type (*p* < 0.05). Especially, the 45S5 BAG-containing experimental groups in this study clearly showed a great increase in pH, both in rate and magnitude, within the immersion time. As soon as 45S5_B specimens are immersed in acid solution, the pH value immediately increases and continues to do so during the 180 min. These patterns are similar to the S53P4_B group. The pH rise of the 45S5F BAG-containing experimental groups was less pronounced compared to other BAG groups. Nevertheless, immersion of 45S5F_B in lactic acid for 180 min resulted in a solution with a neutral pH. This final pH was comparable to the results of BAG_0 and Transbond XT groups, which retain acidic conditions around pH 4.0. Increasing the BAG filler proportion significantly decreased the time to raise the pH from four to 5.5. In particular, 45S5_B took (9.5 ± 1.1) min to reach pH 5.5, which was much faster than (59.0 ± 8.5) min for 45S5_A (*p* < 0.05). However, (9.7 ± 1.5) min for S53P4_B was not significantly different from that of 45S5_B (*p* > 0.05). In comparison, 45S5F_A, S53P4_A, BAG_0 and Transbond XT did not raise the pH to 5.5 until 180 min.

Therefore, it was confirmed that the three types of BAG fillers have an ability to neutralize the acid solution, while the time taken to raise the acidic pH was shorted as the increasing proportion of BAG was added. Ranking by acid neutralizing ability showed 45S5 BAG groups to have the highest effect followed by the S53P4 and 45S5F BAG groups. Transbond XT and BAG_0 were the groups with no neutralizing effect.

### 2.3. Shear Bond Testing and the Adhesive Remnant Index

**Figure 5:**
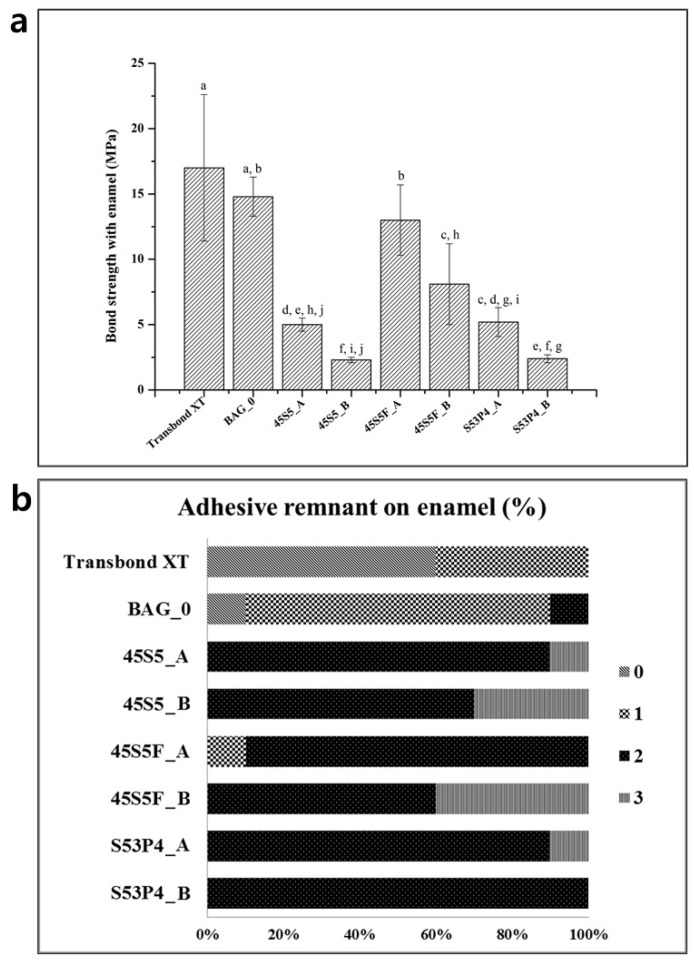
Shear bonding test for experimental orthodontic adhesive. (**a**) Mean and standard deviation for shear bond strength (SBS). The same letters indicate no statically-significant difference (*p* > 0.05); (**b**) Adhesive Remnant Index (ARI) score (% of total) for the debonded enamel surface. ARI scores: 0, no adhesive remaining on tooth; 1, less than 50% adhesive remaining on tooth; 2, more than 50% adhesive remaining on tooth; 3, all adhesive remaining on tooth.

Figure 5a shows the shear bond strength between enamel with the experimental orthodontic adhesive groups (mean ± SD, *n* = 10). The results showed that the Transbond XT and BAG_0 groups resulted in statistically similar shear bond strength (SBS) values (*p* > 0.05). In contrast, the bond strength values of all of the BAG-containing experimental groups were lower than Transbond XT (*p* < 0.05). Furthermore, the results showed a decreasing trend of bond strength with an increasing proportion of each BAG added. Nevertheless, 45S5F_A’s bond strength was not significantly different from BAG_0 (*p* > 0.05). Ranking by the shear bond strength of the BAG-containing experimental groups, 45S5F BAG showed the highest value, followed by S53P4 and 45S5.

**Figure 6:**
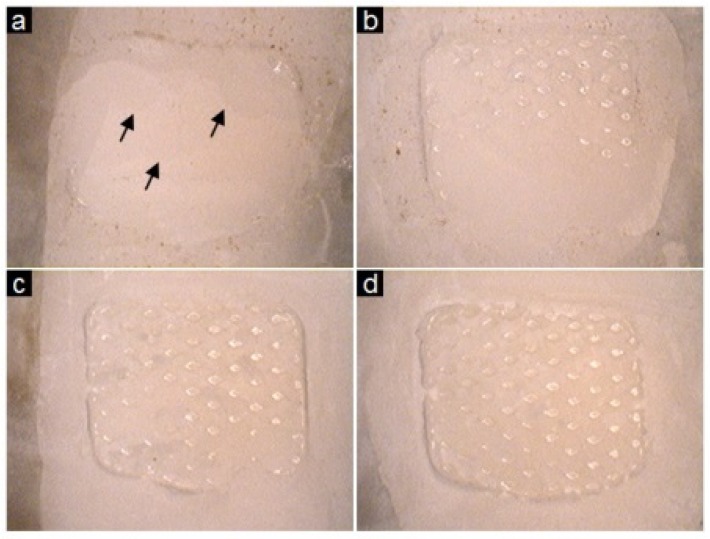
Debonded surfaces after shear bonding test. (**a**) Debonded enamel surface after shear bonding test of Transbond XT. Black arrows indicate cracking of sound enamel; (**b**) Debonded enamel surface after shear bonding test of BAG_0; (**c**) Debonded enamel surface after shear bonding test of 45S5F_A; (**d**) Debonded enamel surface after shear bonding test of 45S5F_B.

Figure 5b shows a comparison of the Adhesive Remnant Index (ARI) scores, which revealed a similar trend among the BAG-containing experimental groups tested. These results revealed that the three types of BAG did not cause changes to the bonding process; whereas Transbond XT groups showed low ARI scores and the enamel surface of the group exhibited evidence of scratching, cracking and loss of sound enamel (Figure 6).

## 3. Discussion

The mechanism for dental caries’ formation is known to take place in the following steps: (1) cariogenic bacteria in the biofilm metabolize acids from fermentable carbohydrate; (2) metabolized acids result in a decrease in the pH, causing demineralization of the tooth; and (3) demineralization is continued, and caries are formed [22]. The adhesion and accumulation of biofilm was shown to be significantly higher at the orthodontic adhesive materials than any parts of the bracket materials during orthodontic treatment [23]. In this regard, reducing the demineralization of enamel near the adhesive is essential in preventing WSLs. Therefore, neutralizing of metabolized acids should be considered for the cariostatic properties of adhesives around the area. The present study compared the potential for utilizing three different novel types of orthodontic adhesives containing BAGs for the prevention of WSLs. Furthermore, the study was aimed to find the ideal composition that maximizes the acid neutralizing properties, while minimizing the bond failure with enamel. Our previous study with 45S5 BAG-containing pit and fissure sealant had shown an acid neutralizing effect in a cariogenic environment [14]. 45S5 BAG particles underwent a series of surface reactions that included the release of soluble alkali ions, such as Na^+^ and Ca^2+^ ions, in aqueous environments, resulting in an increase in the aqueous pH value [24]. Using a similar approach here, our current study showed the acid neutralizing effect to prevent tooth demineralization, such as WSLs. Three different BAG-containing adhesives were evaluated for their acid neutralizing property in a cariogenic environment and were also used to measure the bond strength with bovine tooth. In this study, the demineralization possibilities of the tooth structure due to cariogenic acid challenge were evaluated using acid neutralizing properties. This method can be correlated to demineralization occurrence, since WSLs are areas of mineral loss, which are caused by the dissolution of enamel apatite [25]. Comparison groups in the present study were orthodontic adhesive without BAG and commercial adhesive, Transbond XT. In this study, all BAG-containing groups were significantly acid neutralized compared to control groups. Furthermore, among all of the BAG-containing adhesives, the one with 61.25% of BAG resulted in more of a significant increase of pH compared to the one with 52.5% of BAG (*p* < 0.05), indicating evidence of a greater increase in final pH values as more BAG was added. In addition, the acid neutralizing effect was ranked in the following order: 45S5, S53P4 and 45S5F BAG. It was possible to assume that this is due to their respective chemical compositions. Especially, 45S5 BAG groups demonstrated a superior ability to raise the pH, perhaps due to an increased release of ions and a higher bioactivity rate when compared to the 45S5F BAG [26]. In a previous study, when using a constant amount of glass for each composition, fluoride-containing BAG demonstrates a less pronounced pH rise [27]. This could be explained by ion exchange processes at the glass surface, caused by F^−^ ions that are exchanged with OH^−^ ions. Removing OH^−^ ions from the solution and providing F^−^ ions would result in a buffer effect of alkali ion release, and therefore, the increasing fluoride content in the glass resulted in a less pronounced pH rise than 45S5 BAG [28]. Furthermore, the other study demonstrated that fluoride-containing BAG leads to a slower release of ions in solution during the bioactivity test [29]. Nonetheless, in the current study, the acid neutralizing ability of 45S5F BAG in a short time was shown in a cariogenic environment compared to control groups. The Stephan curve indicated that following a glucose mouth rinse, dental plaque pH decreases to the value corresponding to a cariogenic pH for a minimum of 5 to 20 min. This is followed by a gradual recovery to its starting value, usually over 30 to 60 min [14,30]. Thus, it would be desirable for a specimen to quickly raise the cariogenic pH from four to 5.5 or above in order to help resist dental caries.

Another study showed that S53P4 BAG suspensions resulted in a pH of 11 because of the release of sodium oxide from the glass surface on wetting [11]. In the same manner, in this study, the acid neutralizing of S53P4 has been attributed to its ability to increase the pH in acid solution.

Furthermore, our study compared the effects of the different amounts of BAG fillers on the acid neutralizing ability and the shear bond strength. The BAG_0 and Transbond XT groups containing only silanized dental glass filler had significantly higher shear bond strength than BAG filler-containing groups. According to a previous study, silanization treatment of the filler increased the mechanical properties [31,32]. Therefore, the BAG_0 group has a comparable SBS value to the Transbond XT.

However, the trade-off results showed that decreasing of the BAG fillers decreased the acid neutralizing ability, while shear bond strength was increased, likely due to the hydrophobic silane coating on the dental glass filler (silanized dental glass filler) retarding the access of water to the filler, slowing dissolution [31].

In addition, for our preparatory experiment, SBS values of 70.0% for the BAG groups were very low for orthodontic bracket bonding, while having superior acid neutralizing ability (data not shown). Therefore, based on our preparatory results, we had excluded the 70.0% of BAG groups that only had BAG-containing adhesive as the experimental group due to too low of a bond strength for bracket bonding in orthodontic treatment.

The present study sought to determine the best concentration of BAG as the orthodontic adhesive filler, keeping the balance between the acid neutralizing effect and bonding properties. Under the experimental procedure of the present study, the 45S5F BAG of 61.25% (Group 45S5F_B) in the orthodontic adhesive provided a similar bond strength to the clinically-acceptable bond strength of an orthodontic bracket, which should be at least 5.9–7.8 MPa to permit adequate adhesion [33]. Hence, the novel 45S5F BAG containing the orthodontic adhesive of the present study appeared to yield adequate bond strength with enamel. 45S5F BAG, which has more resistance to dissolution in an aqueous environment than the other BAGs, such as 45S5 and S53P4, with orthodontic adhesive should help maintain the bond strength for the experimental time. Our previous study indicated that the release of the ions according to the dissolution of BAG in an aqueous environment may cause weakening of the mechanical properties of the material [14]. It is also revealed that the ARI in the present study for Transbond XT showed the incidence of enamel cracking during the debonding process. On the other hand, the debonded surfaces of BAG-containing groups showed an undamaged state after shear bonding test. Because of this phenomenon, we should consider the materials to improve the maintenance of clinically-useful bond strength while minimizing the amount of tooth destruction [34].

It is therefore speculated that using 45S5F BAG as the filler of orthodontic adhesive is advantageous because of its acid neutralizing ability and reasonable SBS compared to the control group. The limitation of the current study was the short observation period of the acid neutralizing and bonding properties. In our previous published work, we evaluated the effects on the 45S5 BAG filler for the inhibition of enamel demineralization that occurred over a prolonged period of time (15, 30 and 45 days) in a cariogenic environment. This study showed that increasing the immersion time in a cariogenic environment induced a slightly decreasing trend of resistance to the acid solution [35]. Despite this information, we still shall study a longer duration of exposure for 45S5F and S53P4’ in terms of acid neutralizing ability and bond strength. In conclusion, all three types of BAG in our study may be satisfactory for inhibiting demineralization of enamel, but most of all, 45S5F BAG was recommended for the application to the orthodontic adhesive filler.

## 4. Experimental Section

### 4.1. Preparation of Orthodontic Adhesives Containing BAG

SiO_2_ (Junsei Chemical Co., Tokyo, Japan), Na_2_CO_3_ (Duksan Pure Chemicals Co., Ansan-city, Korea), CaCO_3_ (Samchun Pure Chemicals Co., Pyeongtaek city, Korea), P_2_O_5_ (Sigma-Aldrich, Steinheim, Germany) and CaF_2_ (Sigma-Aldrich, Steinheim, Germany) powders were prepared to make the three different types of glass for the experiments (Table 1). The batch of 45S5 BAG was melted in a platinum crucible at 1400 °C for 1 h and then quenched into a graphite plate mold at room temperature to prevent crystallization. The batches of 45S5F BAG and S53P4 BAG were also melted in a platinum crucible at 1430 °C for 1 h and 1360 °C for 3 h, respectively, using a melt-quench route. Each of three different melt-derived types of BAG was then ground using a fast mill (Ceramic Instruments, Sassuolo, Italy) and not silanized for proper ion release from BAGs in an aqueous environment. To confirm the particle size and morphology of the three different types of BAG, the histograms of the BAGs’ particle size distribution were obtained using the laser scattering particle size distribution analyzer (LA-950, HORIBA, Kyoto, Japan) and a scanning electron microscope (SEM, JEOL JSM-7001F, JEOL Ltd., Tokyo, Japan) with an accelerating voltage of 15.0 kV at 10,000× magnification. Furthermore, the amorphous structures of the three types of BAG powder were confirmed by X-ray diffraction (XRD, Ultima IV, Rigaku, Tokyo, Japan) examination.

A resin matrix was prepared with a mix of 49.5 wt% bisphenol A glycerolate dimethacrylate, 49.5 wt% triethylene glycol dimethacrylate, 0.3 wt% of camphorquinone and 0.6 wt% of 2-(dimethylamino) ethyl methacrylate. All of these raw materials were purchased (Sigma-Aldrich, Steinheim, Germany) and prepared in the lab.

To perform the orthodontic adhesive experimental groups, 45S5 BAG, 45S5F BAG, S53P4 BAG and silanized dental glass filler (NanoFine^®^ NF180, Schott, Landshut, Germany) of (180 ± 30 nm) were added to the resin matrix in different proportions, and commercially available Transbond XT light-cured adhesive paste (3M Unitek, Monrovia, CA, USA) was used as a reference material (Table 2).

### 4.2. Acid Neutralizing Ability

To make the specimens, a stainless steel mold (25 mm × 2 mm × 2 mm) was filled with the uncured experimental orthodontic adhesive, avoiding the formation of air bubbles. All materials were cured by a light-curing unit (Elipar™ S10, 3M ESPE Co., Seefeld, Germany) for 20 s on each side. Then, the cured experimental orthodontic adhesive specimen was separated from the mold. To investigate the neutralizing ability of each group, the specimens were immersed in a lactic acid (Sigma-Aldrich, Steinheim, Germany) solution of pH 4.0, yielding a specimen/solution ratio of 0.14 cm^3^/1 mL, at (25 ± 1) °C [14]. Three specimens (25 mm × 2 mm × 2 mm) were immersed in 2.14 mL of lactic acid solution. As soon as the specimens were submerged in lactic acid solution, time-dependent changes of the pH in acid solution were monitored with a pH electrode (Orion 4 Star, Thermo Fisher Scientific Inc., Ayer Rajah Crescent, Singapore) for 180 min, and data were acquired each minute. Furthermore, the time to raise the pH from 4.0 to 5.5 was evaluated.

### 4.3. Shear Bond Testing and the Adhesive Remnant Index

Seventy bovine incisors were used in this study. A micro motor (Marathon-4, Sae yang Microtech, Korea) and a diamond-coated disk (NTI-Kahla GmbH, Kahla, Germany) were used to separate the crowns from the roots. Separated crowns were embedded with the labial surface in a self-cured acrylic resin (Polycoat, Aekyung, Chungnam, Korea) in cylindrical molds of 20 mm in diameter. After polymerization of the resin, teeth were ground using a polishing machine (Ecomet, Buehler Ltd., Lake Bluff, IL, USA) with 600, 800 and 1200 grit silicon carbide paper (Deerfos, Incheon, Korea), sequentially, to obtain a uniform parallel enamel surface. The enamel was etched with 35% phosphoric acid (Unitek Etching Gel, 3M Unitek, Monrovia, CA, USA) for 30 s, followed by rinsing with distilled water for 15 s and air drying for 10 s until the etched enamel showed a frosty white appearance. After then, liquid primer (Transbond XT primer, 3M Unitek, Monrovia, CA, USA) was applied to the etched enamel and light cured for 10 s. Each experimental orthodontic adhesive paste was applied on the base of the upper central incisor metal bracket (Daeseung medical Co., Seoul, Korea), and the bracket was placed onto the tooth in the center of the enamel surface. To standardize bonding adhesive thickness and pressure, a 1 pound Gilmore needle was held vertically on the bracket while excessive adhesive around the bracket bases was removed with an explorer before polymerization [36]. The light-curing unit was then applied in mesial and distal direction for 10 s on the experimental adhesives between enamel and bracket. After bonding the bracket to the enamel surface, the specimens were then stored in distilled water for 24 h at (37 ± 1) °C before bond strength testing.

A universal testing machine (Instron 5942, Instron, Norwood, MA, USA) was used to debond the bracket from the enamel at a crosshead speed of 0.5 mm/min. A chisel-edged plunger was mounted in the movable crosshead of the testing machine and positioned to apply a shear force on the area between the enamel and experimental orthodontic adhesive interface. An occluso-gingival load was applied at the enamel-adhesive interface. The shear bond strength (SBS) was then calculated by dividing the force required to debond in Newtons by the surface area of the bracket base (12 mm^2^). After the SBS test, all debonded enamel surfaces from the brackets were observed by the same operator using an optical microscope (Hi-Scope, KH-1000, Hirox Co., Ltd., Tokyo, Japan) at 10× magnification, to determine the ARI scores. These scores quantify the remaining adhesive material on the enamel to evaluate the area where the fracture had occurred during the SBS test. The ARI score ranges from 0 to 3 with each score indicating: 0, no amount of adhesive remaining in the enamel; 1, less than half of the adhesive remaining in the enamel; 2, more than half of the adhesive remaining in the enamel; and 3, all of the adhesive remaining in the enamel [37].

### 4.4. Statistical Analysis

The acid neutralizing ability and SBS data were analyzed with one-way ANOVA (PASW 18.0, IBM Co., Chicaco, IL, USA) followed by Tukey’s statistical test. The significance level was fixed at 0.05.

## 5. Conclusions

This study compared the acid neutralizing effect and shear bond strength with enamel of three different types of orthodontic adhesives containing BAGs, and the following conclusions were drawn.

The first null hypothesis, there are no significant differences in the acid neutralizing property among the three different types of BAGs, was rejected. The acid neutralizing ability indicated by the pH change of the acid solution using three types of BAG was shown to be the best for the 45S5, followed by S53P4 and 45S5F. Furthermore, among all of the BAG-containing orthodontic adhesives, the one with 61.25% of BAG showed a significantly greater increase of the pH than the one with 52.5% of BAG.

The second null hypothesis, there are no significant differences in the shear bond strength among the three different types of BAGs, was rejected. The bond strength with enamel that was shown by the shear bonding test using orthodontic adhesive containing three types of BAG indicated that the highest value was from 45S5F, followed by S53P4 and 45S5. Furthermore, among all of the BAG-containing orthodontic adhesives, the one with 61.25% of BAG showed a significantly lower SBS than the one with 52.5% of BAG.

Therefore, based on these results, we can suggest that 45S5F_B is recommended as a promising filler of orthodontic adhesive due to it having clinically-acceptable bond strength and acid neutralizing property for inhibiting WSLs around orthodontic adhesives.

## Figures and Tables

**Table 1:** Three different batches of bioactive glass for the experiments (wt%).

Compositions	SiO_2_	CaCO_3_	Na_2_CO_3_	P_2_O_5_	CaF_2_
45S5	45.0	24.5	24.5	6.0	–
45S5F	45.0	12.5	24.25	6.0	12.25
S53P4	53.0	20.0	23.0	4.0	–

**Table 2:** Weight ratio of filler proportions in the experimental groups (wt%).

Experimental Group	Resin Matrix	Silanized Dental Glass Filler	BAG filler
Transbond XT *	20 to 30	70 to 80	–
BAG_0	30.0	70.0	–
45S5_A	30.0	17.50	52.50
45S5_B	30.0	8.75	61.25
45S5F_A	30.0	17.50	52.50
45S5F_B	30.0	8.75	61.25
S53P4_A	30.0	17.50	52.50
S53P4_B	30.0	8.75	61.25

Note: * Compositions of Transbond XT are stated in the safety data sheet (MSDS) provided by the manufacturers.
